# Agroforestry systems of the lowland alluvial valleys of the Tehuacán-Cuicatlán Biosphere Reserve: an evaluation of their biocultural capacity

**DOI:** 10.1186/1746-4269-11-8

**Published:** 2015-02-19

**Authors:** Mariana Vallejo, Alejandro Casas, Edgar Pérez-Negrón, Ana I Moreno-Calles, Omar Hernández-Ordoñez, Oswaldo Tellez, Patricia Dávila

**Affiliations:** Centro de Investigaciones en Ecosistemas, UNAM campus Morelia, Antigua Carretera a Pátzcuaro No. 8701, Col. Ex-Hacienda de San José de la Huerta, Morelia, 58190 Michoacán Mexico; Escuela Nacional de Estudios Superiores Unidad Morelia, UNAM campus Morelia, Antigua Carretera a Pátzcuaro No. 8701, Col. Ex-Hacienda de San José de la Huerta, Morelia, 58190 Michoacán Mexico; UBIPRO, Facultad de Estudios Superiores Iztacala, UNAM, Avenida de los Baños, s/n, Apartado Postal 54090 (Los Reyes Iztacala), Tlalnepantla, Estado de México

**Keywords:** Agroforestry systems, Biocultural heritage, Biodiversity conservation, Mezquital, Silvicultural management, Sustainable management, Tehuacán Valley, Traditional agriculture

## Abstract

**Background:**

Agroforestry systems (AFS) are valuable production systems that allow concealing benefits provision with conservation of biodiversity and ecosystem services. We analysed AFS of the zone of alluvial valleys of the Tehuacán-Cuicatlán Valley (TCV), Mexico, the most intensive agricultural systems within a region recognized for harbouring one of the most ancient agricultural experience of the New World. We hypothesized that the biodiversity conservation capacity of AFS would be directly related to traditional agricultural features and inversely related to management intensity.

**Methods:**

Agricultural practices, use frequency of machinery and chemical inputs, and proportion of forest and cultivated areas were described in 15 AFS plots in alluvial valleys of the Salado River in three villages of the region. With the information, we constructed a management intensity index and compared among plots and villages. We documented the reasons why people maintain wild plant species and traditional practices. Perennial plant species were sampled in vegetation of AFS (15 plots) and unmanaged forests (12 plots 500 m^2^) in order to compare richness, diversity and other ecological indicators in AFS and forest.

**Results:**

In all studied sites, people combine traditional and intensive agricultural practices. Main agroforestry practices are ground terraces and borders surrounding AFS plots where people maintain vegetation. According to people, the reasons for maintaining shrubs and trees in AFS were in order of importance are: Beauty and shade provision (14% of people), fruit provision (7%), protection against strong wind, and favouring water and soil retention. We recorded 66 species of trees and shrubs in the AFS studied, 81% of them being native species that represent 38% of the perennial plant species recorded in forests sampled. Land tenure and institutions vary among sites but not influenced the actions for maintaining the vegetation cover in AFS. Plant diversity decreased with increasing agricultural intensity.

**Conclusions:**

Maintenance of vegetation cover did not confront markedly with the intensive agricultural practices. It is possible the expansion and enrichment of vegetation in terraces and borders of AFS. Information available on plant species and local techniques is potentially useful for a regional program of biodiversity conservation considering AFS as keystones.

## Background

Alluvial valleys are sites particularly propitious for agriculture, because of their nutrients rich soils, irrigation and flat terrains favouring intensive practices [1, 2]. These ecosystems are also important areas for designing strategies to control floods, watershed recharge, carbon storage, and biodiversity conservation [3]. However, these functions are in high risk, because of the high transformation these ecosystems have experienced. The remaining natural areas are generally degraded and fragmented, and their transformation has affected the hydrological systems, increasing sediments and water contamination [4]. These ecosystems are therefore a priority for conservation worldwide.

Forest loss worries the contemporary societies not only because of the associated biodiversity decline, but also because of the degradation of ecosystem services. Rural peoples are greatly affected, particularly those of tropical areas where communities traditionally depend on the diversity of products provided by forests [5]. For this reason, current science and society direct great efforts in designing systems capable to combine the provision of benefits with biodiversity conservation [6]. For designing management programs it is crucial that decision makers consider opinions of scientists, as well as knowledge and experience of local people that have practiced for long time local ecosystem management [7, 8]. Traditional agriculturalists continually change their natural resources management techniques, influenced by changes in economic, technical, and social variables, and human values [9–12]. In some contexts, such changes may favour biodiversity conservation, but in others, these may influence severe ecosystems degradation. Understanding both situations is thus crucial for constructing knowledge for sustainability.

In rural areas of the tropical zones, indigenous peoples maintain the main reservoirs of traditional agriculture knowledge and techniques [13], as well as germplasm diversity highly important for *in situ* conservation of genetic resources [9]. Because plant resources of forests maintained in these systems depend on human management, a parallel evolution of crops and forest resources through practices modelled by humans can be found [14]. Indigenous communities are recognized because of their ecological knowledge, experience in local ecosystem management, and socio-cultural practices and values that contribute to maintain natural resources [15, 16].

Among agricultural practices carried out by indigenous communities, the agroforestry systems (AFS) are outstanding, since join wild and domesticated plant and animal components [17, 18]. The practice of AFS is a historical tradition in different parts of the world [8, 15], representing a variety of relations between humans and nature involving multiple forms of managing resources [16, 19]. Nearly 1.2 billion people practice AFS in the world [20, 21], México being recognized because of the high diversity of these systems occurring in its territory [22, 23].

AFS conform strategies to maximize in small spaces agriculture, livestock and forest management, combining production of food, fodder, fuel wood and multiple useful products. Some of them provide resources and ecosystem services similar to those provided by forests, such as water infiltration, conservation of wild species habitat and corridors, maintenance of pollinators, seed dispersers, and predators of insects that constitute potential pests, and an outstanding contribution to biodiversity conservation [6, 24]. For these reasons, AFS produce significant benefits to people that directly manage them, as well as to human societies in a wider context; these systems are generally recognized as sustainable management systems [25].

A high variety of AFS have been described; they may be diverse, multi-stratified systems with intimate interrelationships among wild and domesticated components, but also these may be plantations of wood and/or fruit producing trees of few species [8]. Composition and attributes of AFS are determined by the role the components play in people’s economy and environmental values. Among reasons recorded about why people leave standing wild plants in agricultural plots, their usefulness is the most common and it is usually related to the perception people have about the availability of useful plants in forests [26]. Other reasons such as their intrinsic value, aesthetic aspects, ceremonial and religious rituals, and transmission of knowledge on forest among generations have been recorded [5, 27]. Faye *et al*. [28] documented that agriculturalists clearly explain that increasing the number of tree species in plots minimize the risk in the functioning of the whole agricultural system. Maintaining diversified forest cover in small production plots is a subsistence strategy [29], which in turn favours resilience of both, the agricultural system and the household that manage the agricultural system.

It has been widely documented that AFS play an important role in biodiversity conservation at different scales [26, 30–32] and may significantly contribute to ecological restoration [24, 33]. However, since plant species vary in functional features such as dispersion capacity and vulnerability to agricultural activities, biodiversity in different taxonomic groups may respond differently to agricultural intensification [34]. According to [34], species richness of vegetation has a direct correlation with landscape complexity and local management. Numerous AFS not only maintain biodiversity of the forests they derive from but also they may increase the diversity including non-native species [16, 35–37].

However, not all AFS are designed for conservation of native biodiversity [5]. Some intensive AFS systems look for producing commercial value trees, in which conservation and interaction among the species maintained in the system are not considered. For this reason, the design of AFS and reasons for such a design are determinant of the characteristics of the system and their conservation capacity. Main variables to consider for characterizing AFS are: (1) structural and floristic diversity, (2) level of agricultural intensification, (3) features of the original forest system, (4) technical aspects of the system, (5) distance to urban and forest areas [6].

Our study was conducted in the Tehuacán-Cuicatlán Valley (TCV), Mexico, a region recognized for its high biological diversity [38], outstanding human cultural richness [39], with a history of more than 10000 years old, early signs of agriculture [40], and a high richness of ethnobotanical knowledge and plant management techniques [39, 41–43]. In the alluvial valleys of the Salado and Grande Rivers, it is established the main intensive agricultural zone of the region, with irrigation systems, use of machines and chemical inputs [44]. In this zone, peasants are the managers of intensive agriculture through AFS. The predominant original vegetation was and in some parts still is the mezquital forest dominated by *Prosopis laevigata*[45]. In the region, AFS have been studied in different zones to construct a regional diagnosis of systems capacity for conserving biodiversity and provision of resources for household’s wellbeing. The general purpose of these studies is the design of a regional strategy of biodiversity conservation based on AFS. Studies by Moreno-Calles *et al*. [46] in the arid zones and by Vallejo *et al*. [37] in the highlands allow a partial view of the panorama. This study complements the previous efforts.

In our current study, the main questions were how the structure of AFS of the intensive agriculture of the traditional Tehuacán Valley is and how it is related to the management practiced by local communities. Which are the main reasons people maintain these systems and what is their capacity for maintaining biodiversity. We expected that the communities managing the system combine features of intensified agriculture with traditional agroforestry practices. Such combination of techniques could provide important lessons about the current trade-offs about conservation and production. We hypothesized that the traditional techniques favour biodiversity conservation, whereas the modern intensification techniques counterbalance such capacity; however, we expected to identify optimum characteristics of the system for achieving both purposes. Our study therefore aimed to characterize strengths and weaknesses of AFS of the alluvial valleys, in order to identify key aspects for improving their sustainable management.

## Methods

### Study area

**Figure 1:**
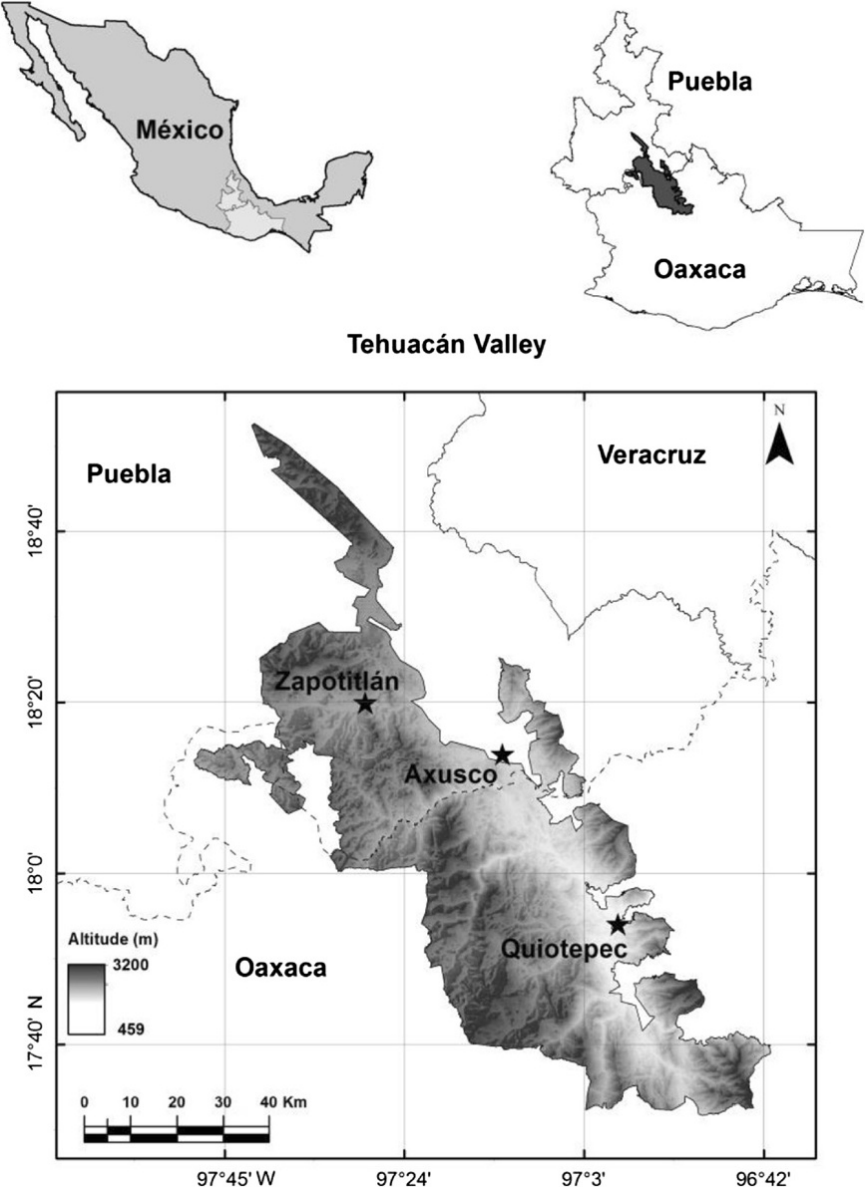
**Study area.** Location of the communities studied in the alluvial valleys of the Salado and Grande Rivers at the Tehuacán-Cuicatlán Biosphere Reserve in Puebla and Oaxaca, central Mexico.

The TCV is located at the southeast of the state of Puebla and the northwest of the state of Oaxaca [47], covering an area of 10,000 km^2^ with a high environmental heterogeneity including 36 vegetation types [45, 48]. Most of the regional territory is semiarid with annual mean precipitation of 300–500 mm [49]. This zone is recognized for its high biodiversity, with more than 3,000 plant species, nearly 400 of them being endemic to the region [38]. It is also culturally diverse, with eight indigenous ethnic groups inhabiting the area [39]. These entire elements make the TCV one of the most important biocultural regions of Mexico (Figure 1).

Our study was conducted in forests and AFS of the lowlands of the region, which is an extent alluvial valley formed by the Salado and Grade rivers, both of them tributaries of the Papaloapan River, one of the greatest rivers of Mexico. The original vegetation in this zone is formed by a combination of thorn-scrub forest, tropical dry forest, and riparian vegetation dominated by the mezquite *Prosopis laevigata*, reason why Valiente-Banuet *et al*. [50] describe them using the term mezquitales. We conducted the study in three areas following the main rivers. One of them is part of the territory of the community of Santiago Quiotepec, Oaxaca, which is inhabited by Mestizo and Cuicatec people. The second area is part of the community of San José Axusco, Puebla, inhabited by Nahua people. The third area is part of the community of Zapotitlán de las Salinas, Puebla, inhabited by Mestizo, Mixtec and Popoloca peoples (Figure 1). In Quiotepec, people are predominantly dedicated to agriculture, cultivating the multi-crop maize system called milpa, complemented by commercialization of lemon and mango; it is located at an elevation of 545 m, where vegetation is tropical dry forest. In Axusco people complement resources from milpa with monetary incomes from intensive cultivation of sugar cane; it is located at elevation of 900 m, where vegetation are thorn and tropical dry forests dominated by mezquite. In Zapotitlán the milpa system is complemented with incomes from the extraction of mineral salt and mining and handicrafts manufactured with onyx; it is located at elevations of 1400 m, with patches of thorn forest and columnar cacti forests dominated by mezquite.

### Study system

In all communities, we studied the milpa system that combines management of maize, beans, squashes, and in some cases chili peppers. In general, the milpa is a small system of nearly one-hectare extent, whose production is for direct consumption by households. We sampled five plots of AFS in each site, in total 15 AFS units. We carried out characterizations at both regional and local levels per community, considering human cultural, technological, and economic aspects related to the management of each AFS plot, as well as ecological and biodiversity conservation issues. For characterizing AFS we mapped each plot, identifying and measuring the area covered by each type of agroforestry practices. Based on these maps we calculated the percentage of vegetation cover of each plot and we censed all species of shrubs and trees occurring in the vegetation patches. We conducted semi-structured interviews to people managing each plot analysed, all of them being the owners of the plot. Interviews focused on five main topics: (1) Physical aspects of the terrain, plot size, use period, land tenure. (2) Agricultural practices, crops managed, fallow periods, patterns of crop rotation, use of fire, instruments, tools and /or machines used, use of agrochemical inputs, irrigation, labour hand invested. (3) Techniques of vegetation management associated to each type of agroforestry practices, reasons why people maintain or remove wild plants, caring actions, and the most valued plant species. (4) Governmental and non-governmental programs enhancing or not AFS, and communitarian rules regulating use and management of forest. (5) Pastoralist practices, how people manage livestock associated to AFS.

The authors state that the study was conducted after the correspondent permit and consent from the authorities of the Biosphere Reserve Tehuacán-Cuicatlán, the local authorities and communitarian meetings in the communities of Quiotpec, Axusco and Zapotitlán de las Salinas, as well as the persons collaborating in the research.

#### Maintenance of shrubs and trees in AFS

Based on information obtained from the interviews, as well as on fieldwork observations, we identified the main reasons why people let standing trees and shrubs in their AFS, as well as the percentages of the more mentioned attributes. We also analysed the reasons why people carry out the different agroforestry practices.

#### Agricultural intensification

Based on the interviews we calculated an index of agriculture intensification, in order to compare the relative state of management intensity of AFS among the communities studied (Table 1). The index is an algorithm that sum indicators of three main components: *use of machinery*, *agrochemical inputs* and *agricultural practices*. For each activity we obtained quantitative values of several indicators and we assigned numerical values for some qualitative variables, in order to standardize and make comparable the information [51]. The component *machinery* included the type of tools used (spade, machete, plough, tractor) at different moments of the agricultural cycle. The component *agrochemicals* included the record of using fertilizers, herbicides and insecticides, their frequency and amounts used. The component *agricultural practices* considered use of fire, frequency and intensity, the number of years practicing agriculture in the plot, the area of the terrain in relation to the practices, the number of times that the land has been cultivated consecutively, the duration of fallow periods, weeding and tilling regimes, irrigation frequency and labour invested. Each component was standardized to percentage 0–100 values, with 300 as the maximum (100%) value. We called this index Intensification Value Index. In addition, in the interviews we included questions about the amounts of maize harvested per agricultural cycle, then dividing the data by the cultivated area to estimate productivity. With this information we calculated the relation between intensification and productivity.

**Table 1:** **Factors included in the index of agriculture intensification**

	Variables	Lower value	Higher value	Values range
***Machinery***	Using machinery	Manual, machete, spade	Plough, Tractor	1 manual, 2 Plough, 3 tractor
	Frecuency of use	No using	All along	0 no, 1 occasionally, 2 all along
***Agrochemical***	Using agrochemical	No using	Using chemical fertilizer, pesticide and herbicide	0 no, 1 organic fertilizer, 2 chemical fertilizer, 3 pestice and herbicide
	Frecuency of use	No using	All along	1 no, 2 all along
***Agricultural practices***	Use of fire	No using	Yes	0 no, 1 yes
	Frequency	No using	All along	0 no, 1 all along
	Intensity	No using	high flame	0 no, 1 high flame
	Number of years practicing agriculture in the plot	1 year	60 years	1 a 60
	Irrigation frequency	No using	All along	0 no, 1 occasionally, 2 all along
	Labour invested	Family	Payment of wages	1 Family, 2 family and support community, 3 family and occasionally payment, 4 all payment

#### Biodiversity conservation

The capacity of biodiversity conservation of AFS was evaluated through vegetation studies, analysing species richness, composition and diversity. We focused our attention on identifying native plant species and estimating the proportion that are maintained in agricultural plots compared with those occurring in the forest from which the AFS derive. We conducted vegetation sampling in 27 plots of 500 m^2^ (50 m × 10 m), subdivided in 100 m^2^ squares (10 m × 10 m); 12 plots sampled in the forest areas and 15 plots in the AFS in each of the communities (Table 2). This is a sampling methods that is conducted at regional level by several research groups in order to make comparable information on vegetation [45, 48]. All individual of shrubs and trees were recorded, measuring their height, two perpendicular diameters of their canopies and, in trees, we also measured the breast height diameter of the trunk (BHD). We collected botanical sampled, and the samples are in the Herbarium of the Centro de Investigaciones en Ecosistemas, UNAM.

**Table 2:** **General characteristics of agricultural plots of the SAF sampled in the study**

Parcels	Land tenure	Lot size	Cultivated land	Sampling
1 Zapotitlán	Communal	4 ha	3 ha	500 m^2^
2 Zapotitlán	Communal	1 ha	0.5 ha	500 m^2^
3 Zapotitlán	Communal	4 ha	3 ha	500 m^2^
4 Zapotitlán	Communal	4 ha	3 ha	500 m^2^
5 Zapotitlán	Communal	3 ha	3 ha	500 m^2^
1 Axusco	Ejidal	1 ha	1 ha	500 m^2^
2 Axusco	Ejidal	3 ha	1 ha	500 m^2^
3 Axusco	Ejidal	1.5 ha	0.5 ha	500 m^2^
4 Axusco	Ejidal	2 ha	1.5 ha	500 m^2^
5 Axusco	Ejidal	2 ha	2 ha	500 m^2^
1 Quiotepec	Private	3 ha	1 ha	500 m^2^
2 Quiotepec	Communal	1 ha	0.5 ha	500 m^2^
3 Quiotepec	Private	2 ha	1 ha	500 m^2^
4 Quiotepec	Private	1 ha	0.5 ha	500 m^2^
5 Quiotepec	Private	1.5 ha	1 ha	500 m^2^

### Data analyses

#### Vegetation

For each plot sampled (in both AFS and forests) we calculated species richness, diversity, species composition, and the el Ecological Importance Value (EIV). In addition, in AFS we calculated structural parameters such as total biomass, frequency of heights and total number of individual plants in order to compare amounts and state of perennial plants inside the agricultural plots. Composition was evaluated by identifying the number of plant families, genera, species, distinguishing those of native species from the total numbers. The Ecological Importance Value (EIV) is a quantitative relation of the relative frequency, density and biomass of each species in the sampled area. Species richness was estimated through the rarefaction method developed by Colwell through the programme *EstimateS*, using the non- parametric estimator Chao [52–54].

Based on Jost *et al*. [55] and using the SPADE program [56], for each site we calculated the true diversity measure; this is analytical approach that has been recognized as the most appropriate for diversity evaluations. We calculated for each site studied the ^1^*D* value (exponential of Shannon’s entropy), ^1^*D* weights each species according to its abundance in the community, and hence, it can be interpreted as the number of ‘common’ species in the community [55, 57].

To compare abundance values between forest and agroforestry sites, we used a generalized linear model (GLM) through *Poisson error* by data counting; we also compared biomass, height and Shannon exponential (continuous data) through linear models [58]. Finally, we compared Shannon exponential values between wild and agroforestry sites, using Student’s *t-*tests, which is useful when there is a single factor and two levels [58]. Before conducting *t*-tests we checked the homogeneity of variance I order to be sure about the validity of the tests.

## Results

### Characterization of AFS and their management

**Figure 2:**
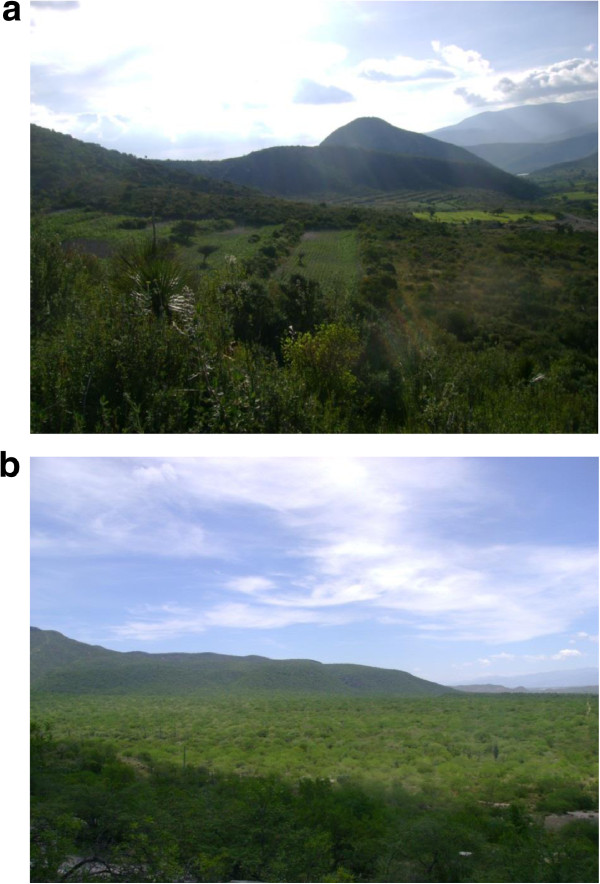
**Aspect of agroforestry systems and forest of the alluvial valley of the Tehacán-Cuicatlán Valley, Mexico. (a)** Plot of agroforestry system showing the ground borders with vegetation forming flat terrains. **(b)** Aspect of the “***mezquital***” thorn forest described by Valiente-Banuet *et al*. [45, 48].

**Figure 3:**
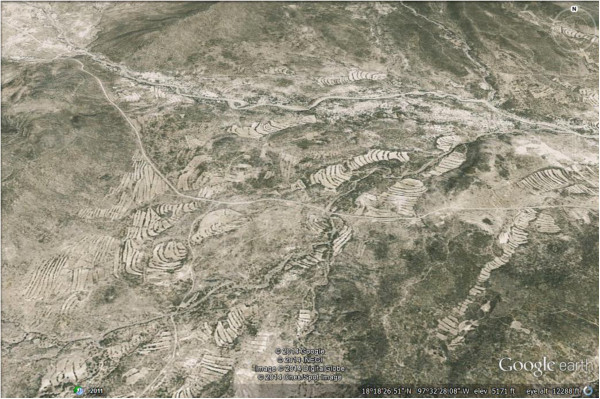
**Aspect of the terraces formed by ground borders stabilized with vegetation cover in the basin of the Salado River and smaller tributaries in Zapotitlán Salinas, Puebla.**

AFS studied have a spatial arrangement according to the specific type of terrain, which are organized in ground terraces or ‘bancales’, which is a pre-Columbian technique practiced in terrains over soft slopes, slightly modifying the surface. This is generally a system directed to retain and protect soils through hedgerows of bushes and ground borders along edges [59, 60]. In this region the technique has the Náhuatl name of ‘metepantle’, meaning “space between agaves” [61], although some variations of the technique use other specific names; for instance, they can be called ‘apantle’ when agaves are absent or ‘melgas’ (term that makes reference to the space destined to cultivation) and ‘estacadas or cabezales’, properly to the ground border. It is just on the ground borders where people maintain wild plant species, sometimes combining with cultivated shrubs and trees (Figure 2). This AFS is so important at the regional level that significantly model the landscape and makes conspicuous the spatial arrangement of terrains in alluvial valleys (Figure 3).

According to the interviews, the establishment of ground terraces helps to manage water from rainfall (retaining it and controlling the run-off causing erosion) as well as from the irrigation system (directing its flow and favouring infiltration). In addition, terraces helps to prevents soil erosion; in both roles, vegetation maintained on the borders plays a central role (Table 3). But maintaining vegetation has more reasons; people mentioned a total of 20 reasons, including utilitarian explanations mainly provision of fruits (7%) and other edible resources (7%), fuel wood (5%), medicines (2%) and fodder for livestock (5%). People also made reference to functions (ecosystem services) such as preventing soil erosion (2%), (make the terrain planner (2%), protection against strong wind (7%), retention of water (7%), provision of shade (14%), barrier of access to domestic animals (2%), as well as attractors of wild animals that may affect the crops (2%). For instance, they mentioned birds, which in wild vegetation find food that otherwise is looked for in the crops. People in addition mentioned reasons related to provision of habitat to other desirable specie (9%); for instance, the edible caterpillar called ‘cuchamá’ the larvae of the butterfly *Paradirphia fumosa*, which are food highly appreciated in the region and has considerable economic value. The larvae ‘cuchamá’ grow associated to the tree ‘manteco’ *Parkinsonia praecox*, which for this and other reasons is particularly appreciated and maintained by people in AFS. Another animal protected in AFS are the edible insects (Hemiptera), ‘cocopache’. Also important is the ‘pitahaya’ *Hylocereus undatus*, associated to mezquite (*Prosopis laevigata*), which produces edible fruits. We also recorded aesthetic reasons (14%) (beauty) for maintaining trees and shrubs, in some areas people call these beautiful plants “wild luxury”, these are the cases of ‘sotolín’ (*Beaucarnea gracilis*), the ‘viejito’ (*Cephalocereus columna-trajani*) and other succulent plants. In addition, people gave ethic and spiritual reasons (“if there is no reason to remove them, why to do it”; the ‘pirul’ *Schinus molle* provides protection against ‘bad spirits’) (2%). Not all reasons are equally important, the most frequently mentioned were those of beauty and shade provision (14%), followed by provision of fruit, protection against strong wind and water retention (7% each) (Table 3).

**Table 3:** **Reasons expressed by people of the communites studied in the Tehuacán Valley for maintaining shrubs and trees in their agroforestry plots**

	Total	Zapotitlán	Axusco	Quiotepec
Reasons	%	%	%	%
Asthetic (beauty)	14	25	7.5	0
Shade	14	15	7.5	18
Maintaining water	7	10	0	9
Windbreaker	7	5	7.5	9
Food (other than fruit)	7	0	15	9
Unnecessary to remove them	7	0	15	9
Fruit	7	0	0	27
Fuel wood	5	10	0	0
Habitat of the edible larvae *cuchamá*	5	10	0	0
Other uses	5	5	7.5	0
Fodder	5	0	7.5	9
Maintaining of soil	2	5	0	0
Habitat of pitahayas	2	5	0	0
Tools	2	0	7.5	0
Ritual	2	0	7.5	0
Atractor of other species	2	0	7.5	0
Rules	2	0	7.5	0
Medicinal	2	0	0	9
Habitat of edible Hemiptera *cocopaches*	2	5	0	0
Making the terrain plainer	2	5	0	0

In each community studied, the reasons for maintaining shrubs and trees in AFS were variable, but two reasons were mentioned in all sites: protection against strong wind and beauty of the plot. Each person mentioned from one to five reasons. In Zapotitlán, people mentioned 11 of the 20 reasons referred to in the whole region, each person mentioned on average four reasons, the most important being beauty of crop field (25% of mentions). In Axusco people mentioned on average 2.6 reasons, the most important being provision of food and ethic motives (15% each). In Quiotepec people mentioned on average 2.2 reasons, the most important being obtaining of fruit (27%, Table 3). However, when asking the question about the most interesting attribute of shrubs and trees maintained, people mentioned specific uses (65%) followed by their size, they prefer big trees (35%).

Trees and shrubs more valued by people interviewed were mezquites (*Prosopis laevigata*), ‘guajes’ (*Leucaena esculenta*), lemon (*Citrus limon*), ‘manteco’ (*Parkinsonia praecox*) and ‘nopales’ (*Opuntia* spp.) (Table 4). *Prosopis laevigata* had the highest record of preference and the highest number of uses. It is valued as protection barrier against strong wind, levelling the terrain, its pods and leaves are good fodder, its wood is very good fuel wood and material for construction and handicrafts. It is particularly good support for growing ‘pitahaya’ (*Hylocereus undatus*), the edible insects ‘cocopaches’ live on this tree, and it provides shade and beauty to the plot.

**Table 4:** **Most important trees according to the mention of people interviewed**

	Todos	Zapotitlán	Axusco	Quiotepec
***Mezquite*** (*Prosopis laevigata*)	13	5	4	4
***Guaje*** (*Leucaena esculenta*)	7	1	4	2
***Lemon*** (*Citrus limon*)	5	1	1	3
***Manteco*** (*Parkinsonia praecox*)	4	3	1	0
***Nopal*** (*Opuntia sp*.)	4	1	3	0

The number of trees and shrubs and vegetation cover maintained in AFS is highly variable. On average, vegetation cover is 12% of agricultural plots. In Zapotitlán the cover was on average 18% while in Axusco it was 5% and in Quiotepec 10%. Reasons mentioned to remove vegetation cover were: “…plants cause obstruction to the pass of tractor and plough”, and “…it is necessary to remove plants growing fast and difficult to control which affect crop growing”. People also mentioned to remove those plants with spines that hurt persons, those that compete with crops, and those determining excessive shade that affects maize growth and that do not provide any other important function to the system.

People prevent problems associated to maintain trees and shrubs through agroforestry practices that allow them getting benefits and reducing negative effects. In general people maintain vegetation in ground borders (75%), and they function as windbreaker barriers, whereas those in limits of the plot function as live fences. Put in these ways vegetation maintained allow free passing to tractors and plough in the cultivated areas. Trees and shrubs should be pruned to control shade, and the resulting material provide fuel wood. In 80% of the plots studied, people extract fuel wood for direct consumption by the households. The species more valued for this purpose are *Prosopis laevigata* and *Parkinsonia praecox*. These important trees are tolerated as isolated trees within the cultivation areas. Other species are usually transplanted from the centre of the plot to the terraces or borders, but not all of them establish successfully after transplantation. The most successful to this action are agaves and cacti. The agroforestry practices mentioned were recorded in all sites studied, the main difference among sites is the total vegetation cover and the reasons to maintain shrubs and trees.

### Land tenure and use rules

In México land tenure may be private, ejidal and communal, and all of these types are represented in the sites studied. However, in Zapotitlán all AFS studied are in communal land, whereas in Axusco in ejidal land and in Quiotepec in private areas. Cutting trees, even those in sites of the production area is under regulation, requiring permit from local authorities. In Zapotitlán the regulations are particularly strict, the study zone is part of a protected area and sanctions are supervised by the Mexican Ministry of Environment as well as by local authorities. In Axusco the Ejidal authority or Comisariado is the one in charge of authorizing or not the vegetation removal, whereas in Quiotepec it is the Communal authority (Comisariado), but people may remove trees without permit in their private land. Sanctions are generally economic but in Zapotitlán people that disobey may be jailed.

People (nearly 47% of interviewees) said that PROCAMPO (a governmental programme in charge of supporting agricultural production) through economic incentives proportional to cultivated land, penalizes the areas maintaining vegetation, which is not considered to be ‘productive’. Contrarily, other governmental programs from SEMARNAT (Mexican Ministry of Environment) or CONANP (National Commission of Protected Areas) enhance reforestation or planting trees in plots. In nearly 33% of the plots sampled we recorded these programs but these were unsuccessful. In Zapotitlán these institutions promoted agaves and *Parkinsonia praecox* but the mortality of plants was high.

### Vegetation

On average, we recorded 13 species of trees and shrubs per plot of AFS. However, variation is very high, ranging from 3 to 33 species per plot. In Zapotitán we recorded the highest number of woody species (17.5 ± 5) per plot, whereas in Quiotepec and Axusco we recorded 11.6 ± 3 and 9.2 ± 1.15 woody species per plot, respectively.

#### Floristic composition

**Figure 4:**
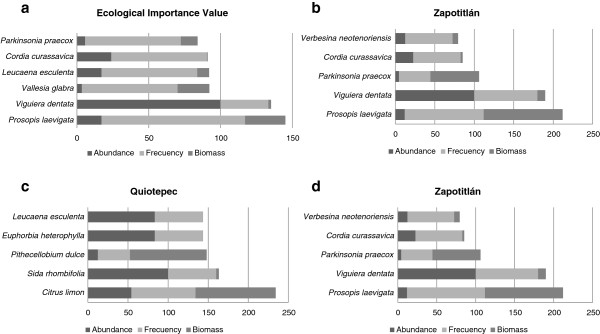
**Ecological Importance Value indexes calculated for the most important plant species in agroforestry systems of the sites studied (a) General results at regional level, (b) Zapotitlán (c) Quiotepec, (d) Axusco.**

Through the vegetation sampling we recorded a total of 66 species of trees and shrubs in the AFS studied. These species belong to 30 plant families and 49 genera (Table 5). The most represented plant families are Cactaceae with 13 species, and Fabaceae with 12 species. Most species of trees and shrubs recorded (81%) in AFS are native species, which represent approximately 38% of the perennial plant species recorded in the vegetation sampling of forests of the alluvial valleys studied. According to their Ecological Importance Value, The most important species in AFS are *Prosopis laevigata, Viguiera dentata, Vallesia glabra, Leucaena esculenta, Cordia curassavica,* and *Parkinsonia praecox* (Figure 4). *Prosopis laevigata* is the most frequent and *Viguiera dentata* the most abundant.

**Table 5:** **Checklist of trees and shrubs species recorded in Agroforestry systems (AFS) and the natural forest**

Voucher	Families	Species	Forest	AFS
MVR254	Acanthaceae	*Justicia candicans* (Nees) L.D. Benson		**x**
MVR261	Amaranthaceae	*Iresine* sp.	**x**	
Photo	Anacardiaceae	*Amphipterygium adstringens* (Schltdl.) Standl.	**x**	
		*Mangifera indica* L.		**x**
MVR263		*Pseudosmodingium multifolium* Rose	**x**	
MVR259		*Schinus molle* L.	**x**	**x**
Photo	Annonaceae	*Annona cherimola* Mill.		**x**
MVR296	Apocynaceae	*Vallesia glabra* (Cav.) Link	**x**	**x**
		*Plumeria rubra* L.	**x**	
Photo	Asparagaceae	*Agave karwinskii* Zucc.	**x**	
Photo		*Agave macroacantha* Zucc.	**x**	
Photo		*Agave marmorata* Roezl	**x**	**x**
Photo		*Agave potatorum* Zucc.	**x**	
Photo		*Agave salmiana* Otto ex Salm-Dyck		**x**
Photo		*Agave* sp.		**x**
MVR320	Asteraceae	*Gymnosperma glutinosum* (Spreng.) Less.		**x**
MVR325		*Montanoa grandiflora* DC.		**x**
MVR313		*Sanvitalia fruticosa* Hemsl.	**x**	
MVR229		*Verbesina neotenoriensis* B.L. Turner	**x**	**x**
MVR265		*Viguiera dentata* (Cav.) Spreng.	**x**	**x**
MVR307		Morfo1		**x**
Photo	Bignoniaceae	*Tecoma stans* (L.) Juss. ex Kunth		**x**
Photo	Boraginaceae	*Cordia curassavica* (Jacq.) Roem. & Schult.	**x**	**x**
MVR224		*Cordia stellata* Greenm.	**x**	
Photo	Bromeliaceae	*Hechtia glomerata* Zucc.	**x**	
Photo		*Hechtia sphaeroblasta* B.L. Rob.	**x**	**x**
Photo	Burseraceae	*Bursera aloexylon* (Schiede ex Schltdl.) Engl.	**x**	
Photo		*Bursera aptera* Ramirez	**x**	
MVR218		*Bursera cuneata* (Schltdl.) Engl.	**x**	
MVR299		*Bursera fagaroides* (Kunth) Engl.	**x**	
Photo		*Bursera morelensis* Ramírez	**x**	
Photo		*Bursera schlechtendalii* Engl.	**x**	**x**
Photo		*Bursera submoniliformis* Engl.	**x**	
Photo	Cactaceae	*Cephalocereus columna-trajani* (Karw. ex Pfeiff.) K. Schum.	**x**	**x**
Photo		*Coryphantha pallida* Britton & Rose	**x**	**x**
Photo		*Cylindropuntia leptocaulis* (DC.) F.M. Knuth	**x**	
Photo		*Echinocactus platyacanthus* Link & Otto	**x**	**x**
Photo		*Escontria chiotilla* (F.A.C. Weber) Rose	**x**	
Photo		*Ferocactus latispinus* (Haw.) Britton & Rose	**x**	**x**
Photo		*Mammillaria carnea* Zucc. ex Pfeiff.	**x**	**x**
Photo		*Mammillaria haageana* Pfeiff.	**x**	**x**
Photo		*Mammillaria sphacelata* Mart.	**x**	**x**
Photo		*Mammillaria* sp.	**x**	
Photo		*Myrtillocactus geometrizans* (Mart. ex Pfeiff.) Console	**x**	
Photo		*Neobuxbaumia tetetzo* (J.M. Coult.) Backeb.	**x**	
Photo		*Opuntia depressa* Rose	**x**	
Photo		*Opuntia ficus-indica* (L.) Mill.		**x**
Photo		*Opuntia pilifera* F.A.C. Weber	**x**	**x**
Photo		*Opuntia pubescens* J.C. Wendl. ex Pfeiff.	**x**	**x**
Photo		*Opuntia pumila* Rose	**x**	
Photo		*Opuntia* sp.	**x**	**x**
Photo		*Pachycereus hollianus* (F.A.C. Weber) Buxb.	**x**	**x**
Photo		*Pachycereus weberi* (J.M. Coult.) Backeb.	**x**	
Photo		*Polaskia chichipe* (Gosselin) Backeb.	**x**	
Photo		*Pilosocereus chrysacanthus* (F.A.C. Weber ex Schum.) Byles & G.D. Rowley	**x**	
Photo		*Stenocereus pruinosus* (Otto ex Pfeiff.) Buxb.	**x**	
Photo		*Stenocereus stellatus* (Pfeiff.) Riccob.	**x**	
Photo		*Peniocereus viperinus* (F.A.C. Weber) Buxb.		**x**
MVR315	Cannabaceae	*Celtis pallida* Torr.	**x**	**x**
MVR305	Cannaceae	*Canna indica* L.	**x**	**x**
MVR211	Capparaceae	*Capparis incana* Kunth	**x**	
Photo	Convolvulaceae	*Ipomoea arborescens* (Humb. & Bonpl. ex Willd.) G. Don	**x**	**x**
Photo	Ebenaceae	*Diospyros digyna* Jacq.	**x**	**x**
MVR322	Euphorbiaceae	*Argythamnia guatemalensis* Müll. Arg.	**x**	
MVR219		*Cnidoscolus tehuacanensis* Breckon	**x**	
MVR221		*Croton glabellus* L.	**x**	
Photo		*Croton alamosanus* Rose	**x**	
MVR295		*Croton* sp.	**x**	
MVR223		*Euphorbia graminea Jacq.*	**x**	
MVR287		*Euphorbia heterophylla* L.	**x**	**x**
MVR324		*Euphorbia verticillata* Desf.	**x**	
MVR385		*Euphorbia* sp.		**x**
Photo		*Jatropha rzedowskii* J. Jiménez Ram.	**x**	
Photo		*Mabea occidentalis* Benth.	**x**	
MVR230		*Manihot pauciflora* Brandegee	**x**	
Photo		*Pedilanthus tehuacanus* Brandegee	**x**	
Photo		*Ricinus communis* L.		**x**
MVR215	Fabaceae	*Acacia angustifolia* (Lam.) Desf.	**x**	**x**
MVR228		*Acacia cochliacantha* Humb. & Bonpl. ex Willd.	**x**	
Photo		*Acacia coulteri* Benth.	**x**	
MVR298		*Acacia farnesiana* (L.) Willd.	**x**	**x**
MVR209		*Acacia pringlei* Rose	**x**	
MVR290		*Acacia* sp.		**x**
MVR236		*Caesalpinia melanadenia* (Rose) Standl.	**x**	
MVR271		*Cercidium praecox* (Ruiz & Pav. ex Hook.) Harms	**x**	
MVR308		*Dalea carthagenensis* (Jacq.) J.F. Macbr.	**x**	**x**
MVR269		*Dalea* sp.	**x**	
MVR245		*Dalea* sp.	**x**	
MVR213		*Dalea* sp.	**x**	
MVR260		*Hymenaea courbaril* L.	**x**	**x**
MVR298		*Leucaena esculenta* (Moc. & Sessé ex DC.) Benth.		**x**
MVR232		*Mimosa luisana* Brandegee	**x**	**x**
MVR309		*Mimosa polyantha* Benth	**x**	
MVR269		*Mimosa* sp.		**x**
Photo		*Parkinsonia praecox* (Ruiz & Pav. ex Hook.) Hawkins	**x**	**x**
Photo		*Pithecellobium dulce* (Roxb.) Benth.		**x**
Photo		*Prosopis laevigata* (Humb. & Bonpl. ex Willd.) M.C. Johnst.	**x**	**x**
MVR234		*Senna wislizeni* (A. Gray) H.S. Irwin & Barneby	**x**	
Photo		*Vachellia constricta* (Benth.) Seigler & Ebinger	**x**	**x**
Photo	Fouquieriaceae	*Fouquieria formosa* Kunth	**x**	
Photo	Hernandiaceae	*Gyrocarpus mocinoi* Espejo	**x**	
Photo	Lauraceae	*Persea americana* Mill.		**x**
MVR331	Loasaceae	*Mentzelia hispida* Willd.	**x**	
Photo	Lythraceae	*Punica granatum* L.		**x**
MVR231	Malpighiaceae	*Echinopterys eglandulosa* (A. Juss.) Small	**x**	
MVR240		*Galphimia glauca* Cav.	**x**	**x**
MVR216	Malvaceae	*Ayenia mexicana* Turcz.	**x**	
MVR297		*Ceiba aesculifolia* (Kunth) Britten & Baker f.	**x**	
MVR220		*Herissantia crispa* (L.) Brizicky	**x**	
MVR222		*Melochia tomentosa* L.	**x**	**x**
MVR280		*Sida rhombifolia* L.	**x**	**x**
Photo	Meliaceae	*Cedrela odorata* L.	**x**	**x**
Photo	Myrtaceae	*Psidium guajava L.*		**x**
MVR244	Phytolaccaceae	*Rivina humilis* L.		**x**
MVR212	Primulaceae	*Jacquinia seleriana* Urb. & Loes. ex Mez	**x**	
MVR303	Rhamnaceae	*Ziziphus amole* (Sessé & Moc.) M.C. Johnst.	**x**	
Photo		*Karwinskia mollis* Schltdl.	**x**	
MVR261	Rubiaceae	*Randia* sp.		**x**
Photo	Rutaceae	*Citrus limon* (L.) Osbeck		**x**
MVR284	Sapotaceae	*Sideroxylon capiri* (A. DC.) Pittier	**x**	
Photo		*Sideroxylon occidentale* (Hemsl.) T.D. Penn.	**x**	
MVR289		*Sideroxylon palmeri* (Rose) T.D. Penn.		**x**
Photo	Simaroubaceae	*Castela tortuosa* Liebm.	**x**	**x**
MVR253	Solanaceae	*Capsicum annum* L.		**x**
MVR306		*Solanum tridynamum* Dunal	**x**	**x**
MVR286		*Solanum nigrum* L		**x**
MVR266	Verbenaceae	*Lantana achyranthifolia* Desf.	**x**	**x**
MVR268		*Lantana camara* L.	**x**	
Photo		*Lippia graveolens* Kunth.	**x**	**x**

In AFS of Zapotitlán we recorded 48 species, the most important (with the highest EIV) being *Prosopis laevigata* and *Viguiera dentata*, whereas in Quiotepec we recorded 26 species (the highest EIV recorded in *Citrus limon* and *Sida rhombifolia*) and in Axusco 9 species (the highest EIV recorded in *Leucaena esculenta* and *Sideroxylon palmeri* (Figure 4).

#### Species richness and diversity

**Figure 5:**
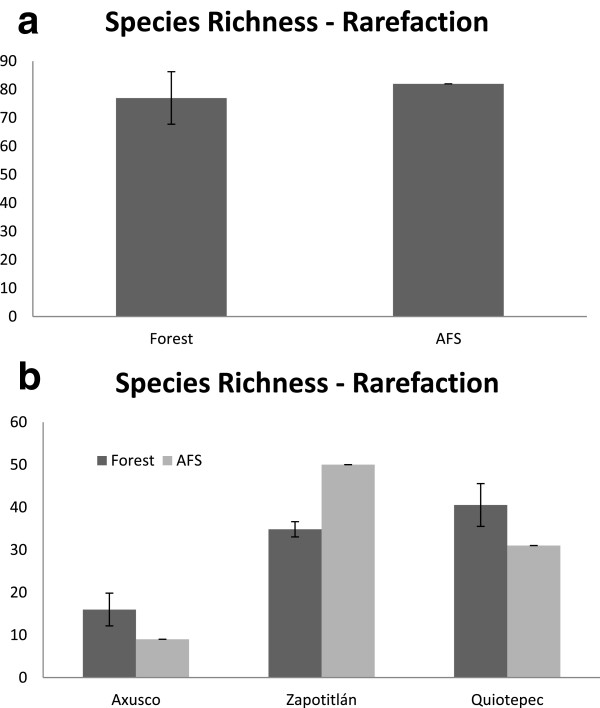
**Plant species richness calculated in agroforestry systems and forest systems of the alluvial valley of the Tehuacán-Cuicatlán Biosphere Reserve through the method of rarefaction. (a)** General comparison **(b)** Comparison of systems in the sites studied.

**Figure 6:**
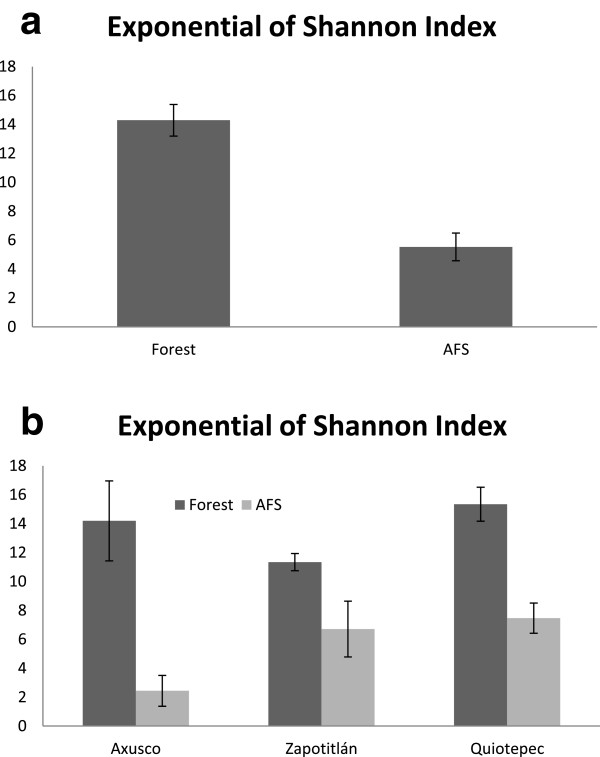
**Plant species diversity calculated in agroforestry systems and forest systems of the alluvial valley of the Tehuacán-Cuicatlán Biosphere Reserve through the exponential Shannon index. (a)** General comparison **(b)** Comparison of systems in the sites studied.

AFS maintain a species richness similar to wild forests, without significant differences, which confirms their important capacity for conserving native biodiversity. The rarefaction plot of species richness (Figure 5) shows that AFS has a relatively higher number of species of trees and shrubs than in forests. But this general pattern changes among sites. In Axusco and Quiotepec the wild forest have significantly higher richness of trees and shrubs than AFS, whereas in Zapotitlán AFS have significantly higher species richness than forests (Figure 5). However, the Shannon exponential index value differs statically between wild forest and AFS (*t* = 6.0387, *p* = 2.62E-06), AFS having significantly lower diversity than forests (Figure 6). Loss of diversity is particularly drastic in the community of Axusco, and although we found differences between Axusco (*t* = 4.33, *p* = 0.003) and Quitepec (*t* = 4.915 *p* = 0.0082), decreasing of diversity in AFS is considerable in Zapotitlán as well as in Quiotepec.

#### Vegetation structure

**Figure 7:**
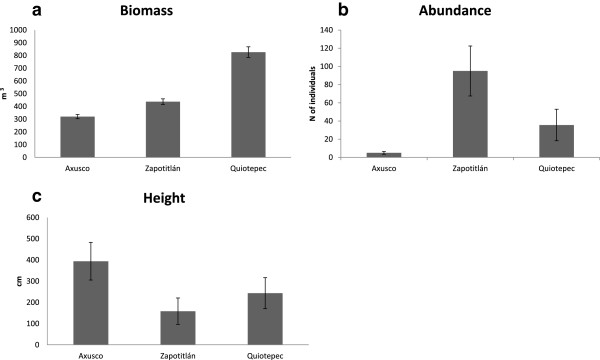
**Vegetation structure parameters comparing agroforestry systems among the sites studied. (a)** Biomass, **(b)** Abundance measured as the number **(c)** Average height of the perennial plants with the AFS.

AFS in Quiotepec have higher biomass than AFS of the other sites but differences are not significant (*biom. F*_13_ = 0.0001, *p* = 0.99; ^1^*D. F*_13_ = 4.017, *p* = 0.06; Figure 7). AFS with the higher number of trees and shrubs were those of Zapotitlán (95 individual woody plants per plot on average). It was followed by Quiotepec (on average 35 individuals of trees and shrubs per plot) and Axusco, where the number of trees and shrubs drastically decreased compared with the other sites (on average five individulas per plot; *ind. F*_13_ = 12.05, *p* = 0.00414, Figure 7). Woody plants in plots of Axusco are significantly taller (on average 4 m tall *h. F*_13_ = 5.21, *p* = 0.039) compared with those in plots of Quiotepec and Zapotitlán (Figure 7).

### Production system

AFS in the sites studied are mainly dedicated to cultivation of milpa (maize, beans, squashes, chilli pepper), together with some fruit-producing trees (mainly lemon, sapodilla and mango) and other horticultural crops such as watermelon and melon in irrigated plots. The milpa products are directly consumed by households whereas fruits are commercialized. Corn (varieties ‘criollo’*,* ‘rojo’, ‘azul’, and ‘híbrido’), beans (*Phaseolus vulgaris* varieties ‘enredador’, ‘flor de mayo’, ‘mosquito’, ‘blanco’, ‘delgado’, ‘mazateco’, ‘negro’, and ‘rojo’) and squashes (*Cucurbita pepo* or ‘calabaza de castilla’, *C. argyrosperma* or ‘calabaza acamotada’ and *C. moschata* or ‘calabaza deapenita’) are grown. We found on average three crop species per plot, but some of them have six crop species (in Zapotitlán 2.5, in Axusco 3, and in Quiotepec 3). No significant differences were identified in this aspect among sites.

We found that nearly 80% of the agricultural plots sampled had irrigation; all agriculturalists make use of tractor for tilling the earth, some of them alternating with plough. The agricultural cycle is from three to four months, in Quiotepec people practicing two cycles of cultivation per year, leaving land in fallow only two months, whereas in Zapotitlán and Axusco people practice one single cultivation cycle per year leaving land in fallow 7 to 8 months. To maintain the soil fertility, people make use of organic inputs, mainly goat dung and leaves; in nearly 60% of the plots sampled people make use of chemical fertilizers. For controlling pests of animals, nearly 60% of people interviewed said to add chemical insecticides whereas the rest said not to make use of any type of pest control. In Axusco all people interviewed said to make use of chemicals for controlling larvae affecting maize. Control of weeds is mainly conducted manually, only 14% people interviewed (all of them from Axusco) said to make use of herbicides.

**Figure 8:**
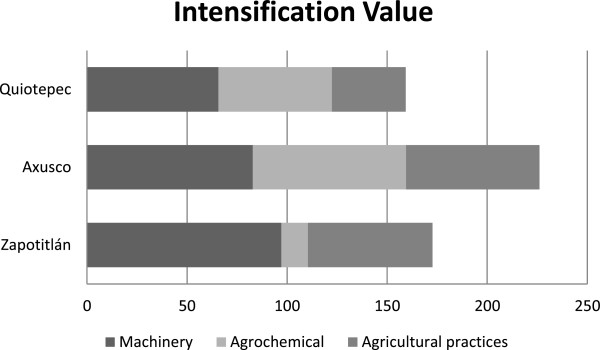
**Intensification Value with the three components analysed:** ***Machinery, Agrochemical*** **and** ***Agricultural practices,*** **comparing agroforestry systems among the sites studied.**

Calculations of *Intensification Value* (Figure 8) indicated that the community of Axusco had the highest values. In this community people make use of machinery more frequently, the higher use of agrochemical inputs was recorded there and agricultural practices are in general more intensive than in the other sites studied (*e. g.* longer use of land in relation to the fallow period, more frequent use of fire). Axusco is followed by Zapotitlán where people make use of tractor combined with plough, but agrochemicals are practically non-used, and the agricultural practices are intense; they have used the land in consecutive cycles for long time. Quiotepec had the lowest values of intensification since use of machinery is less frequent than in the other sites, and although people make use of chemicals this is much less frequent and in lower amounts than in Axusco. In Quiotepec people practice two agricultural cycles per year but they started this use pattern until recently, after land was long time in fallow. Production was generally similar in all sites studied. In Axusco people harvest on average 720 ± 49 kg of maize per ha, whereas in Quiotepec 640 ± 25 kg/ha and in Zapotitlán 588 ± 23 kg / ha.

Livestock raising was markedly different among sites. In Zapotitlán people do not raise domestic animals in the plots of AFS, whereas in Quiotepec 80% of the plots are used for feeding animals during the dry season (mainly maize straw and remaining weeds) and in Axusco 60% of the plots have a similar situation. However, in general few animals are maintained in AFS plots (on average two oxen, one donkey and one horse), in Quiotepec the owner of one plot maintain in his AFS plot 30 goats.

## Discussion

AFS of alluvial valleys studied maintain traditional technological elements combined with modern intensifier agricultural techniques. These are probably the most interesting features for discussing how much these approaches are viable to interact for constructing sustainable productive agricultural systems more effective for satisfying the need of preserving biodiversity and provision of goods and services to society. The ground terraces were described by archaeologists to have existed in the Tehuacán Valley and Oaxaca, Central Mexico with antiquities between 3,000 to 2,000 years before present [62]. Terraces have traditionally been associated to soil and water management, prevention of soil erosion, forms of social organization and diverse cultural aspects [60], all of which are current aspects documented in this study. This ancient technique has changed throughout time, we know that presence of *Agave* spp. in the ground terraces and borders was more important in the past [23], as they are still important in other zones of the Tehuacán Valley. Displacement of agaves and predominance of *Prosopis laevigata* and other species may be associated to decreasing use of agaves for extracting ‘pulque’ (its fermented sap) and the priority to have fuel wood available. The system therefore exists and species composition may be adjusted according to the changing needs and priorities.

The dynamics of the system makes necessary to pay attention to who manage the system and socio-cultural changes occurring in their households, what are the changing reasons and how these influence decisions for maintaining wild plants in the system. Moreno-Calles *et al*. [23] found that in general for AFS of Mexico numerous studies have reported utilitarian aspects as the main reason motivating the decision to maintain wild plants in the system. These authors found that such interest may be expressed in the fact that the proportion of useful species existing in natural forests may increase in AFS (These authors reported an increase of useful species from 75% to 96% in AFS of arid zones). Those studies have identified that among the main uses of components of AFS are fodder and shade, roles that are consistent in the cases analysed in our current study. However, beauty is a particularly relevant reason in the systems of our study.

Not all wild species in AFS have the same cultural value. In the cases studied the most cultural important species are those with the highest Ecological Importance Value (EIV) like *Prosopis laevigata*, which has eight different uses, it is highly appreciated by people and has a high EIV. It is pertinent to indicate that the EIV is an effect of the cultural importance. Another example is *Beaucarnea gracilis*, which has high aesthetic value (it is considered beautiful) as well as culturally (there were parties dedicated to this plant). People let standing these species because they are interested on them, the contrary interpretation (these plants are culturally important because they are abundant) is incorrect.

Capacity of biodiversity conservation is relatively lower than that of other systems studied in the region. In AFS of temperate forest of the highlands, we [37] recorded on average 46% of capacity whereas in columnar cacti forests Moreno-Calles *et al*. [46] recorded up 70% of capacity. Anyway, these systems have features of high intensive management (more than those others studied in the region) and have the capacity of maintaining 38% of native trees and shrubs. We also identified that AFS of the zone studied the diversity decreases more pronouncedly than AFS in temperate forests, and even more than AFS in columnar cacti forests which have a high capacity to maintain plant diversity [36] did not identify significant differences in diversity of AFS and columnar cacti forests.

In terms of structure, the systems varied among the sites studied. In Zapotitlán AFS are more abundant in terms of number of individuals but have lower biomass than in Quiotepec. This pattern can be explained because in Zapotitlán shrubs and small trees are particularly abundant, whereas in Quiotepec people prefer to maintain big trees and even bigger in Axusco, where few individuals are let standing but most of them are big trees.

The production system maintains features of traditional management combined with use of machinery and chemical inputs. These features contribute to make these systems more productive than those of the columnar cacti forests [46] and those of temperate forests in highlands [37]. But corn yields are not impressive (less than one ton per hectare) and although systems differ in the intensity value their differences in production are not significant. Which allows question the real need of the intensive use of machinery and chemical inputs, which determine a higher investment of energy not proportional to the production obtained, making thus the system more inefficient. It is outstanding that even when features of intensive management are present, the AFS studied maintain an important capacity of biodiversity conservation. Contrasting the sites analysed allows seeing that intensive management does not require removing vegetation cover and that, therefore, it is possible and necessary reinforcing the effort for increasing richness and diversity of native plant species in the terraces and ground borders of this system. Natural ecosystems in the sites studied are similar; therefore, the checklist of species reported, as well as others that have been reported in AFS of the neighbouring columnar cacti forests [46] may be the basis for enriching the plant cover of the systems studied. Such species enrichment is not only desirable but technically possible. The regional experience for managing and cultivating native plant species has for the moment identified nearly 300 plant species [42], many of them viable to be used in the alluvial valleys.

Enriching and increasing plant cover in AFS may be a priority strategy promoted by the authorities of the Biosphere Reserve Tehuacán-Cuicatlán, as well as local authorities of communities. Ethnobotanical studies in the region have inventoried nearly 1600 useful plant species, nearly 90% of them being part of the regional forests [39, 41]. There are now conditions to starting a regional program with the purposes of expanding and enriching plant cover of AFS, with the pertinent local species and local techniques. Our studies show that this goal is not confronted with needs of increasing agricultural production. And, for the contrary, that program would contribute to maintain not only plat diversity, but also the associated diversity [63] of animals and insects that find in these microenvironments, important bridges with broader fragments provided by secondary vegetation patches and forest areas. Biodiversity conservation has traditionally been seen as opposed to land use, but AFS reveal that the trade-off is not necessarily true. Conserving biodiversity at regional level should consider biodiversity conservation at landscape level in particular zones. The areas reported in this study, as well as those studied by [36, 37, 46, 64] provide information and techniques that could be adopted by regional authorities for a program on biodiversity conservation considering the productive systems.

Land tenure in Mexico is particularly important for constructing agreements, regulations and institutions. Our study found that the sites studied had the three different regimes (private, comunal and ejidal). We found that collective systems of property have higher capacity to regulate the amount of vegetation cover, but we did not find a concrete influence of this situation on the amount of vegetation cover. In Zapotitlán, because the whole territory is part of the Biosphere Reserve, the authorities of the Reserve control external rules that not always are well considered by local people, but it undoubtedly has contributed to promotion of conservation values and actions. These actions could be enhanced at regional level.

Governmental programs like Procampo do not help in promoting expansion of vegetation cover in AFS. The authorities of the Biosphere Reserve could have an active negotiation with that Federal programme in order to coordinate efforts of the different governmental programmes. However, it is important to mention that Procampo like other governmental programs, including the authorities of the Biosphere Reserve, not always have had the sensibility to include local people in participatory processes to design actions for concealing production and biodiversity conservation. This is a great challenge, and both local people and authorities as well as authorities of the Biosphere Reserve, may be supported by researchers that have generated information like the current and other referred studies for the Tehuacán-Cuicatlán Valley, one of the most important areas of biocultural heritage in Mexico.

The Tehuacán Valley is an eminent arid zone of Mexico, but the agricultural system studied is located in the ‘oasis’ zone provided by the rivers Salado and Grande. Therefore, the comparison of the systems studied should be made with other similar systems of Mexico and the World. Few studies have been published with a similar approach in similar environmental contexts. For nstance, it has been documented that in the Saharan desert traditional agriculture has been maintained throughout time in the oases, where are commonly practiced ancient techniques such as maintenance of trees resistant to soil salinity and favoring Keeling humidity, shade and providing fruits [65]. In Mexico, in the Sonoran Desert, Nabhan [14] documented the traditional agricultural techniques practiced by the Papago people, who have conserved the oases of their territories and have developed a complex system of biotic interactions. This author identified eight plant associations and various agroforestry practices including living fences and windbreaker barriers, as well as high levels of diversity of trees, birds and mammals. The Papago have modified the landscape geomorphology through terraces, channels and flood zones 14,17]. In the Mezquital Valley in central Mexico, the Ñañhú people have constructed terraces and borders to manage water and sediments to improve soil and humidity for crops. Particularly important for these purposes are agave which in addition provide other multiple uses such as food, beverages, and fibers [17].

In the arid zones of the World numerous human cultures have interacted with the difficult conditions of these zones for thousands of years, and a significant amount of knowledge and techniques have been developed [17, 65], which are all crucial at present for designing the future. Investigating trees and shrubs associated to crops may provide valuable information for improving the AFS, conserving biodiversity and supporting techniques for restoring disturbed areas of arid zones [33, 65].

## Conclusions

Information resulting from this study allow confirming the role of AFS as systems able to provide goods and other benefits at the same time that conserving biodiversity and ecosystem services. The AFS studied are the most intensive in the TCV and however, are able to make compatible intensive agricultural practices with biodiversity conservation. Intensive practices should be technically reviewed since they appear to be inefficient and promote contamination. The current practices are compatible with strategies for increasing and diversifying vegetation cover in ground terraces and borders. Local species and management techniques documented for the region make possible such a strategy with high potential benefit.

## Authors’ information

MV postgraduate student at the Centro de Investigaciones en Ecosistemas (CIEco), UNAM. AC full time researchers at CIEco, UNAM. EPN academic technician at CIEco, UNAM. AIMC researcher at the Escuela Nacional de Estudios Superiores, Morelia, UNAM. OHO postgraduate student at the Centro de Investigaciones en Ecosistemas (CIEco), UNAM. OT and PD researchers at the UBIPRO, FES Iztacala, UNAM.

## References

[1] Ryan R, Erickson D, De Young R (2003). Farmers’ motivations for adopting conservation practices along riparian zones in a Mid-western agricultural watershed. J Environ Plan Manage.

[2] Flores A, Castillo A, Sánchez-Matías M, Maass M (2014). Local values and decisions: views and constraints for riparian management in western Mexico. Knowl Manag Aquat Ecosyst.

[3] Frey GE, Mercer DE, Cubbage FW, Abt RC (2010). Economic potential of agroforestry and forestry in the lower Mississippi alluvial valley with incentive programs and carbon payments. South J Appl Forest.

[4] Frey GE, Mercer DE, Cubbage FW, Abt RC (2013). A real options model to assess the role of flexibility in forestry and agroforestry adoption and disadoption in the lower Mississippi alluvial valley. Agr Econ.

[5] Dawson IK, Guariguata MR, Loo J, Weber Ard Lengkeek JC, Bush D, Cornelius J, Guarino L, Kindt R, Orwa C, Russell J, Jamnadass R (2013). What is the relevance of smallholders’ agroforestry systems for conserving tropical tree species and genetic diversity in circa situm, in situ and ex situ settings? A review. Biodivers Conserv.

[6] Jose S (2012). Agroforestry for conserving and enhancing biodiversity. Agrofor Syst.

[7] Grumbine RE (1994). What is ecosystem management?. Cons Biol.

[8] Ajijur Rahman S, Faizar Rahman M, Sunderland T (2014). Increasing tree cover in degrading landscapes: ‘Integration’ and ‘Intensification’ of smallholder forest culture in the alutilla valley, matiranga, Bangladesh. Small-scale For.

[9] Altieri M, Nicholls C (2000). Serie de textos básicos para la formación ambiental. Programa de las naciones unidas para el medio ambiente. Teoría y práctica para una agricultura sostenible.

[10] McFarlane BL, Boxall PC (2000). Factors influencing forest values and attitudes of two stakeholder groups: the case of the Foothills model forest, Alberta, Canada. Soc Nat Resour.

[11] Tindall DB (2003). Social values and the contingent nature of public opinion, attitudes, and preferences about forests. For Chronicle.

[12] Gadd ME (2005). Conservation outside of parks: attitudes of local people in Laikipia, Kenya. Environ Conserv.

[13] Altieri MA, Toledo VM (2011). The agroecological revolution in Latin America: rescuing nature, ensuring food sovereignty and empowering peasants. J Peasant Stud.

[14] Nabhan GP, Amadeo MR, Reichhardt KL, Mellink E, Hutchinson CF (1982). Papago influences on habitat and biotic diversity: quitovac oasis ethnoecology. J Ethnobiol.

[15] Altieri MA (2004). Linking ecologists and traditional farmers in the search for sustainable agriculture. Front Ecol Environ.

[16] Nandy S, Kumar DA (2013). Comparing tree diversity and population structure between a traditional agroforestry system and natural forests of Barak valley, Northeast India. Int J Biodivers Sci Ecosyst Serv Manag.

[17] Moreno-Calles A, García-Luna V, Casas A, Toledo VM, Vallejo M, Santos-Fita D, Camou-Guerrero A (2014). La Etnoagroforestería: el estudio de los sistemas agroforestales tradicionales de México. Etnobiología.

[18] Kyndt T, Assogbadjo AE, Hardy OJ, Glele-Kakaï R, Sinsin B, Damme PV, Gheysen G (2009). Spatial genetic structuring of baobab (*Adansonia digitata*, Malvaceae) in the traditional agroforestry systems of West Africa. Am J Bot.

[19] Wiersum KF (1997). Indigenous exploitation and management of tropical forest resources: an evolutionary continuum in forest-people interaction. Agri Ecosyst Environ.

[20] *World Agroforestry Centre*. http://www.worldagroforestrycentre.org

[21] Zomer RJ, Trabucco A, Coe R, Place F (2009). Trees on farm: analysis of global extent and geographical patterns of agroforestry.

[22] Wilken G (1977). Integrating forest and small-scale farm systems in Middle America. Agro-Ecosystems.

[23] Moreno-Calles AI, Toledo VM, Casas A (2013). Los sistemas agroforestales tradicionales de México: una aproximación biocultural. Bot Sci.

[24] Dosskey MG, Bentrup G, Schoeneberger M (2012). A role for agroforestry in forest restoration in the lower Mississippi alluvial valley. J Forest.

[25] Gao J, Barberi C, Valdivia C (2014). A socio-demographic examination of the perceived benefits of agroforestry. Agrofor Syst.

[26] Assogbadjo AE, Glèlè Kakaï R, Vodouhê FG, Djagoun CAMS, Codjia JTC, Sinsin B (2012). Biodiversity and socioeconomic factors supporting farmers’ choice of wild edible trees in the agroforestry systems of Benin (West Africa). For Policy Econ.

[27] Ansong M, Røskaft E (2011). Determinants of attitudes of primary stakeholders towardsforest conservation management: a case study of Subri Forest Reserve, Ghana. Int J Biodivers Sci Ecosyst Serv Manag.

[28] Faye MD, Weber JC, Abasse TA, Boureima M, Larwanou M, Bationo AB, Diallo BO, Sigue H, Dakouo J-M, Samake O, Sonogo Diaite D (2011). Farmers’ preferences for tree functions and species in the West African Sahel. For Trees Livelihoods.

[29] Van Oudenhoven APE, de Groot R (2011). Editorial: ecological and social factors influencing biodiversity management at different scales. Int J Biodivers Sci Ecosyst Serv Manag.

[30] Acharya KP (2006). Linking trees on farms with biodiversity conservation in subsistence farming systems in Nepal. Biodivers Conserv.

[31] McNeely JA, Schroth G (2006). Agroforestry and biodiversity conservation—traditional practices, present dynamics, and lessons for the future. Biodivers Conserv.

[32] Kabir ME, Webb EL (2008). Can homegardens conserve biodiversity in Bangladesh?. Biotropica.

[33] Moreno-Calles AI, Casas A (2010). Agroforestry systems: restoration of semiarid zones in the Tehuacán Valley Central Mexico. Ecol Restor.

[34] Gonthier DJ, Ennis KK, Farinas S, Hsieh H, Iverson AL, Batáry P, Rudolphi J, Tscharntke T, Cardinale BJ, Perfecto I (2014). Biodiversity conservation in agriculture requires a multi-scale approach. Proc R Soc B.

[35] Schroth G, da Fonseca GAB, Harvey CA, Gascon C, Vasconcelos HL, Izac AMN (2004). Agroforestry and biodiversity conservation in tropical landscapes.

[36] Moreno-Calles A, Casas A, García-Frapolli E, Torres-García I (2012). Traditional agroforestry systems of multi-crop “milpa” and “chichipera” cactus forest in the arid Tehuaca´n Valley, Mexico: their management and role in people’s subsistence. Agrofor Syst.

[37] Vallejo M, Casas A, Blancas J, Moreno-Calles AI, Solís L, Rangel-Landa S, Dávila P, Téllez O (2014). Agroforestry systems in the highlands of the Tehuacán Valley, Mexico: indigenous cultures and biodiversity conservation. Agrofor Syst.

[38] Dávila P, Arizmendi MC, Valiente-Banuet A, Villaseñor JL, Casas A, Lira R (2002). Biological diversity in the Tehuacán-Cuicatlán Valley, Mexico. Biodivers Conserv.

[39] Casas A, Valiente-Banuet A, Viveros JL, Caballero J (2001). Plant resources of the Tehuacán Valley, México. Econ Bot.

[40] MacNeish RS, Byers DS (1967). A summary of subsistence. The prehistory of the tehuacán valley: enviroment and subsistence. vo1.1.

[41] Lira R, Casas A, Rosas-López R, Paredes-Flores M, Rangel-Landa S, Solís L, Torres I, Dávila P (2009). Traditional knowledge and useful plant richness in the Tehuacán-Cuicatlán, México. Econ Bot.

[42] Blancas J, Casas A, Rangel-Landa S, Moreno-Calles A, Torres I, Pérez-Negrón E, Solís L, Delgado-Lemus A, Parra F, Arellanes Y, Caballero J, Cortés L, Lira R, Dávila P (2010). Plant management in the tehuacán-cuicatlán Valley, mexico. Econ Bot.

[43] Blancas J, Casas A, Pérez-Salicrup D, Caballero J, Vega E (2013). Ecological and socio-cultural factors influencing plant management in Náhuatl communities of the Tehuacán Valley Mexico. J Ethnobiol Ethnomed.

[44] CONANP (2013). Programa de manejo reserva de la biosfera tehuacán-cuicatlán.

[45] Valiente-Banuet A, Solís L, Dávila P, Arizmendi MC, Silva PC, Ortega-Ramírez J, Treviño CJ, Rangel-Landa S, Casas A (2009). Guía de la vegetación del Valle de Tehuacan-Cuicatlán.

[46] Moreno-Calles A, Casas A, Blancas J, Torres I, Rangel-Landa S, Pérez-Negrón E, Caballero J, Masera O, García-Barrios L (2010). Agroforestry systems and biodiversity conservation in arid zones: the case of the Tehuacán-Cuicatlán Valley, Central México. Agrofor Syst.

[47] Rzedowski J, Vegetación de México (1978). Vegetación de México.

[48] Valiente-Banuet A, Casas A, Dávila P, Arizmendi MC, Villaseñor JL, Ortega-Ramírez J (2000). La vegetación del Valle de Tehuacán-Cuicatlán. Bol Soc Bot Méx.

[49] García E (1988). Modificaciones al sistema de clasificación climática de Koppen.

[50] Valiente-Banuet A, Arizmendi MC, Rojas-Martínez A, Casas A, Godínez-Alvarez H, Silva C, Dávila-Aranda P, Flemming T, Valiente-Banuet A (2002). Biotic interactions and population dynamics of columnar cacti. Columnar cacti and their mutualists: evolution, ecology and conservation.

[51] Trilleras J (2008). Análisis Socio-ecológico del manejo, degradación y restauración del bosque tropical seco de la región de Chamela-Cuixmala, México. Master Thesis. Posgrado en Ciencias Biológicas.

[52] Colwell RK, Coddington JA (1994). Estimating terrestrial biodiversity through extrapolation. Phil Trans Royal Soc.

[53] Gotelli NJ, Colwell RK (2001). Quantifying biodiversity: procedures and pitfalls in the measurement and comparison of species richness. Ecol Lett.

[54] Colwell RK (2006). EstimateS: statistical estimation of species richness and shared species from samples. Version 8.

[55] Jost L (2006). Entropy and diversity. Oikos.

[56] Chao A, Shen T-J (2010). Program SPADE (Species Prediction and Diversity Estimation).

[57] Jost L (2010). The relation between evenness and diversity. Diversity.

[58] Crawley MJ (2007). The R Book.

[59] Rojas-Rabiela T, Coord R-RT (1991). Agricultura prehispánica. La agricultura En tierras mexicanas desde Sus orígenes hasta nuestros días.

[60] González-Jácome A (2003). Ambiente y cultura en la agricultura tradicional de México, casos y perspectivas. Anales de Antropología.

[61] Whitmore TM, Tuner BL (2001). Cultivated landscapes of middle America on the Eve of conquest.

[62] Donkin RA (1979). Agricultural terracing in the aboriginal new world.

[63] Perfecto I, Vandermeer J (2008). Biodiversity conservation in tropical agroecosystems. Ann N Y Acad Sci.

[64] Larios C, Casas A, Vallejo M, Moreno-Calles AI, Blancas J (2013). Plant management and biodiversity conservation in Náhuatl homegardens of the Tehuacán Valley Mexico. J Ethnobiol Ethnomed.

[65] Nabhan GP (2007). Agrobiodiversity change in a Saharan Desert Oasis, 1919–2006: historic shifts in Tasiwit (Berber) and Bedouin crop inventories of Siwa, Egypt. Econ Bot.

